# *Trichinella spiralis* dipeptidyl peptidase 1 suppressed macrophage cytotoxicity by promoting M2 polarization via the STAT6/PPARγ pathway

**DOI:** 10.1186/s13567-023-01209-2

**Published:** 2023-09-13

**Authors:** Shu Wei Yan, Ru Zhang, Xin Guo, Bo Ning Wang, Shao Rong Long, Ruo Dan Liu, Zhong Quan Wang, Jing Cui

**Affiliations:** Department of Parasitology, Medical College, Zhengzhou University, Zhengzhou, 450052 China

**Keywords:** *Trichinella spiralis*, dipeptidyl peptidase 1, macrophage, M2 polarization, ADCC

## Abstract

*Trichinella spiralis* dipeptidyl peptidase 1 (TsDPP1), or cysteine cathepsin C, is a secretory protein that is highly expressed during the infective larvae and adult worm stages in the intestines. The aim of this study was to investigate the mechanism by which recombinant TsDPP1 (rTsDPP1) activates macrophages M2 polarization and decreases macrophage cytotoxicity to kill newborn larvae via ADCC. RAW264.7 macrophages and murine peritoneal macrophages were used in this study. The results of the immunofluorescence test (IFT) and confocal microscopy showed that rTsDPP1 specifically bound to macrophages, and the binding site was localized on the cell membrane. rTsDPP1 activated macrophage M2 polarization, as demonstrated by high expression levels of Arg1 (M2 marker) and M2-related genes (IL-10, TGF-β, CD206 and Arg1) and high numbers of CD206^+^ macrophages. Furthermore, the expression levels of p-STAT6, STAT6 and PPARγ were obviously increased in rTsDPP1-treated macrophages, which were evidently abrogated by using a STAT6 inhibitor (AS1517499) and PPARγ antagonist (GW9662). The results indicated that rTsDPP1 promoted macrophage M2 polarization through the STAT6/PPARγ pathway. Griess reaction results revealed that rTsDPP1 suppressed LPS-induced NO production in macrophages. qPCR and flow cytometry results showed that rTsDPP1 downregulated the expression of FcγR I (CD64) in macrophages. The ability of ADCC to kill newborn larvae was significantly decreased in rTsDPP1-treated macrophages, but AS1517499 and GW9662 restored its killing capacity. Our results demonstrated that rTsDPP1 induced macrophage M2 polarization, upregulated the expression of anti-inflammatory cytokines, and inhibited macrophage-mediated ADCC via activation of the STAT6/PPARγ pathway, which is beneficial to the parasitism and immune evasion of this nematode.

## Introduction

*Trichinella spiralis* is a parasitic nematode that is distributed worldwide. It can infect a variety of mammals and human beings and cause trichinellosis [1]. In China, eight human trichinellosis outbreaks with 479 cases and 2 deaths were reported from 2009 to 2020, and the principal source of infection was pork from domestic pigs [2]. *T. spiralis* muscle larvae (ML) are released in the stomach after infected meat is digested by gastric fluid and develop into intestinal infective larvae (IIL) following activation by bile and gut contents [3]. The IIL migrate into the gut epithelia and develop into adult worms (AWs) after they moult four times at 31 h post-infection (hpi) [4]. After copulation in the small intestine, female adults deposit newborn larvae (NBL), which pass into the venules and lymphatic vessels and then spread throughout the body via blood circulation finally, they invade skeletal muscles and develop into encapsulated ML [5]. *Trichinella* infection elicits a substantial humoral and cellular immune response [6, 7], but ML residing in skeletal muscle capsules can survive from 1 to 2 years to 10–15 years based on the species of the host and even up to 30 years in humans without any major harm [8]. The long survival of *T. spiralis* in the host is likely related to its immunomodulation and immune escape [9].

*Trichinella spiralis* is a unique parasitic nematode in which both the AW and larvae occupy two different intracellular niches within the same host. Although *T. spiralis* infection triggers an intense host immune response, it can modulate the parasite-specific immune response to ensure the survival of both the host and parasite [10, 11]. In the process of *T. spiralis* infection, the excretory-secretory antigens (ESA) from the parasite play a principal role in establishing parasitism and modulating the immune response; they provide an anti-inflammatory milieu and maintain homeostasis [12]. The ESA from *T. spiralis* IIL, AW and ML contain some functional antigens, and they might be important potential immunomodulators in the host immune response [13–15]. In the early stage of *Trichinella* infection, multiple immune cell types are activated in the host to mainly generate a Th1 immune response that produces large amounts of inflammatory cytokines. As the infection progresses, the dominant immune response is switched from Th1 to Th2 to alleviate the tissue damage caused by the immune response through immune regulation and immunosuppression. The immunomodulatory and inhibitory effects are beneficial for the control of inflammation and pathological lesions resulting from *T. spiralis* infection and for its survival in the host [16].

Macrophages, as a kind of immune cell, play a variety of roles in phagocytosis, cellular immunity and molecular immunology [17]. In helminth infection, macrophages are activated into different inflammatory states, including classically activated macrophages (M1) induced by lipopolysaccharide (LPS) and IFN-γ and alternatively activated macrophages (M2) induced by IL-4 and IL-13. M1 macrophages have proinflammatory effects and are involved in the immune response to bacterial and intracellular pathogen infection. In contrast, M2-type macrophages have anti-inflammatory effects and functions in angiogenesis and wound healing, and M2 macrophages are also associated with Th2 reactions, such as antihelminthic immunity, asthma, and allergies. M1-type macrophages secrete proinflammatory cytokines (TNF-α, IL-1β and IL-6) to participate in the immune response and immune surveillance. M2-type macrophages play a key role in suppressing the immune response by inducing the secretion of inhibitory cytokines, such as IL-4, IL-10 and TGF-β [18].

Antibody-dependent cell-mediated cytotoxicity (ADCC) exerts a pivotal effect on protective immunity against *Trichinella* infection, and ADCC kills and destroys larvae, especially NBL [19, 20]. Macrophages, as the main effector cells of ADCC, exert their killing functions by binding their surface Fc receptor (FcR) to the Fc fragment of anti-*Trichinella* IgG antibodies. Moreover, activated macrophages produce proinflammatory cytokines (e.g., TNF-α and IFN-γ) to enhance their phagocytic function. Activated macrophages also secrete some killing molecules, such as nitric oxide (NO) and reactive oxygen species (ROS), to kill larvae [21, 22]. Recently, some studies have shown that *T. spiralis* infection or its ESA regulates the host’s immune response and mainly induces a Th2-type immune response, which secretes IL-4 and IL-13. These cytokines also activate macrophages and induce macrophage polarization towards M2 [23, 24].

Cathepsin C, also known as dipeptidyl peptidase 1 (DPP1), is a cysteine cathepsin with the unique functions of cleaving peptides and degrading macromolecular proteins. Previous studies showed that cathepsin C1 (CPC1) is crucial for *Toxoplasma gondii* growth and proliferation in host cells. CPC1 protein inhibited NF-κB activation and downregulated the expression of IL-1, IL-6, IL-12 and TNF-α. CPC1 might play an important role in the immune evasion of *T. gondii* [25]. A novel dipeptidyl peptidase 1 from *Trichinella spiralis* (TsDPP1; Gene bank: XP_003379334.1) has been identified in our laboratory; TsDPP1 was highly expressed at the IIL and AW stages, primarily located at the cuticle, stichosome and embryos of this parasite, and it was a secretory protein. Recombinant TsDPP1 (rTsDPP1) had the hydrolysing activity of natural DPP1. rTsDPP1 promoted the larval invasion of intestinal epithelial cells (IECs), whereas anti-rTsDPP1 serum and RNAi inhibited larval invasion of IECs, suggesting that TsDPP1 might be a potential target for an anti-*Trichinella* vaccine. However, the role of TsDPP1 in immune regulation and immune evasion during *T. spiralis* infection has not been reported in the literature.

In this study, RAW264.7 and mouse peritoneal macrophages were used to investigate the ability and mechanism of rTsDPP1 to activate macrophage polarization and kill NBL via ADCC. The results will reveal the regulatory effect of TsDPP1 on macrophage immune function in the process of *T. spiralis* infection and provide a basis for elucidating the host immune evasion of *T. spiralis*.

## Materials and methods

### Animals, parasites and cells

Eight-week-old female BALB/c mice were purchased from the Experimental Animal Center of Zhengzhou University. The animal experiment in this study was performed under the principle of National Guidelines for Experimental Animal Welfare (Minister of Science and Technology, People’s Republic of China, 2006). The animal experiment protocol in this study was approved by the Life Science Ethics Committee of Zhengzhou University (No. ZZUIRB GZR 2019 − 0544). The *T. spiralis* (ISS534) parasite used in this research was obtained from an infected pig in Henan Province of China and maintained by serial passage in BALB/c mice [26]. RAW264.7 macrophages were purchased from the Cell Bank of the Chinese Academy of Sciences, and the cells were incubated at 37 °C in 5% CO_2_ with DMEM containing 10% foetal bovine serum, 100 U/mL penicillin and 100 µg/mL streptomycin [27].

### Preparation of *T. spiralis* adult worm ESA

The ML were recovered from infected murine muscle tissues 42 days post-infection (dpi) by the artificial digestion method as previously described [28]. In brief, *T. spiralis-*infected mice were sacrificed, and the murine carcass was digested with 1% pepsin and acidified water. The mice were orally infected with ML (4000 larvae per mouse). At 6 dpi, the AWs were collected from the intestine by incubating the intestine in 0.9% saline at 37 °C for 2 h. Following washes with saline, 6-day AWs were cultured at 5000 worms/mL in RPMI-1640 (Solarbio, Beijing, China) supplemented with 200 U penicillin/mL and 200 μg  streptomycin/mL at 37 °C and 5% CO_2_ for 18 h. The NBL was collected, and culture medium containing AW ESA was concentrated by using a centrifugal filter (Millipore Amicon Ultra15, NMWL: 3000, USA) [29].

### Preparation of rTsDPP1 and anti-rTsDPP1 polyclonal antibodies

The recombinant plasmid pET-32a/TsDPP1 was induced using 1 mM IPTG at 25 °C for 6 h and expressed in the prokaryotic expression system *E. coli* BL21 (DE3). rTsDPP1 was purified using a Ni–NTA His-tag affinity kit (Sangon Biotech., Shanghai, China) as previously described [30]. Purified rTsDPP1 was used to immunize mice to generate polyclonal antibodies. Briefly, 6-week-old mice were subcutaneously injected with 20 µg of rTsDPP1 emulsified with complete Freund’s adjuvant, followed by three boost immunizations with 20 µg of rTsDPP1 emulsified with incomplete Freund’s adjuvant at a 14-day interval. Seven days after the final immunization, blood was collected from the tail vein, and immune sera were collected [8].

### Preparation of murine peritoneal macrophages

Murine peritoneal macrophages were prepared as previously described [4]. Mice were euthanized and soaked in 75% alcohol for 3–5 s. The mice were intraperitoneally injected with 5 mL of prechilled PBS, and the peritoneal wall was simultaneously pressed for 10 min. Under sterile conditions, the intraperitoneal fluid was aspirated and centrifuged at 250 *g* for 10 min at 4 °C. Peritoneal exudate cells (PEC) were adjusted to 5 × 10^6^ cells/mL with DMEM containing 10% foetal bovine serum (HyClone, Logan, Utah, USA), 100 U/mL penicillin and 100 µg/mL streptomycin. After incubation at 37 °C and 5% CO_2_ for 4 h, the cells were washed 2–3 times with DMEM to remove unattached cells, and purified peritoneal macrophages were obtained [31, 32].

### CCK-8 assay

The effect of rTsDPP1 on RAW264.7 macrophage cellular viability was determined using a Cell Counting Kit-8 assay (CCK-8; Solarbio, Beijing, China) [33, 34]. Briefly, the cells were seeded in a 96-well plate (approximately 1 × 10^3^ cells per well) and cultured under the same conditions as described above. rTsDPP1, recombinant thioredoxin (TRX) tag protein and AW ESA were added into the medium and cocultured with cells for 48 h. Then, 10 µL CCK-8 solutions were added to each well and incubated for another 2 h. The absorbance at 450 nm was measured using a multimode reader (SpectraMax i3X; Molecular Devices, USA). Cell viability was presented as the cell survival rate according to the following formulas: cell survival rate = (OD values of test group – OD values of blank control)/(OD values of PBS control group – OD values of blank control) × 100%.

### In vitro stimulation of RAW264.7 and peritoneal macrophages with rTsDPP1

RAW264.7 and peritoneal macrophages were cultivated in the presence of various stimulating factors. rTsDPP1, TRX tag protein and *T. spiralis* AW ESA (20 µg/mL) were added to the medium, LPS (200 ng/mL) and IL-4 (20 ng/mL) were used as macrophage M1/M2 polarization positive controls, and PBS was used as a negative control [35]. After incubation at 37 °C in 5% CO_2_ for diverse times, the cells were washed with PBS and harvested, and total RNAs and soluble proteins from the above-stimulated cells were prepared for qPCR and Western blotting analysis, respectively [5, 12].

### Immunofluorescence test (IFT)

IFT was performed to investigate the binding of rTsDPP1 and RAW264.7 macrophages as previously described [5, 36]. Briefly, macrophages were cultured on a cover glass in a 6-well plate and cultured until the cells reached over 80% confluence. After washing with PBS three times, the cells were fixed with 4% paraformaldehyde for 20 min at room temperature, incubated with 20 µg/mL proteins (rTsDPP1, AW ESA and TRX tag protein) at 37 °C for 2 h, blocked with 5% goat serum at 37 °C for 1 h, and then incubated with 1:10 dilutions of different serum (anti-rTsDPP1 serum, infection serum and preimmune serum) at 37 °C for 1 h. Alexa Fluor 488-conjugated anti-mouse IgG (1:100; Abways, Shanghai, China) served as the secondary antibody, and 4′,6-diamidino-2-phenylindole (DAPI) was used to dye the cell nucleus. The fluorescence signal was observed under fluorescence and confocal microscopy (Olympus, Tokyo, Japan) [37].

### Western blotting analysis

Soluble cellular proteins were prepared from induced RAW264.7 and peritoneal macrophages using ice-cold cell lysis buffer (Beyotime, Shanghai, China) containing 1 mM phenylmethylsulfonyl fluoride (PMSF). The cell protein concentration was measured using a BCA assay kit (Solarbio, Beijing, China) [38, 39]. Cell proteins were separated by 10% SDS–PAGE, transferred onto polyvinylidene difluoride (PVDF) membranes (Millipore, USA), blocked with 5% skim milk in Tris-buffered saline (TBS) containing 0.05% Tween 20 (TBST) for 2 h, and then incubated with the following primary antibodies in TBST overnight at 4 °C: anti-iNOS antibody (1:10 000; Abcam, London, UK), anti-STAT6 antibody (1:1000; Proteintech, Rosement, USA), anti-phosphorylated (p)-STAT6 (Tyr641) antibody (1:1000; Abmart, Shanghai, China), anti-PPARγ antibody (1:1000; Proteintech, USA), anti-Arg1 antibody (1:1000; Proteintech) and anti-tubulin antibody (1:5000; Abcam). After washing with PBST, the membranes were incubated with HRP-conjugated IgG (1:5000; Southern Biotech., USA) at room temperature for 1 h and visualized by Omni-ECL^Tm^ reagents (Epizyme, Shanghai, China) using a chemiluminescent gel imaging system (Tanon 5200, Shanghai, China). The relative intensities of each protein band were analysed using ImageJ software (National Institutes of Health, Bethesda, Maryland, USA) [24, 40, 41].

### Real-time quantitative PCR (qPCR)

Total RNA was extracted from RAW264.7 cells and peritoneal macrophages by TRIzol reagent (Invitrogen, USA) and quantified by determining the absorbance at 260 nm. A cDNA synthesis kit (Takara, Tokyo, Japan) was used to reverse-transcribe mRNA to cDNA, and all qPCRs were performed in triplicate using the SYBR Green PCR master mix (Takara, Tokyo, Japan) in the ABI Prism 7500 Fast Sequence Detection System (Applied Biosystems, USA) [42, 43]. The sequences of primers used for PCR amplification are shown in Table 1 [44, 45]. Fold expression of target gene expression was calculated with the 2^−ΔΔCt^ method by normalization to the internal control GAPDH gene [46].

**Table 1:** **Specific primer sequences of macrophage markers and cytokines for qPCR analysis**

Gene	Forwards (5′-3′)	Reverse (5′-3′)
GAPDH	GGTTGTCTCCTGCGACTTCA	TGGTCCAGGGTTTCTTACTCC
iNOS	TGGAGCCAGTTGTGGATTGTC	GGTCGTAATGTCCAGGAAGTAG
TNF-α	GGTGCCTATGTCTCAGCCTCTT	GCCATAGAACTGATGAGAGGGAG
IL-1β	TGGACCTTCCAGGATGAGGACA	GTTCATCTCGGAGCCTGTAGTG
IL-6	TACCACTTCACAAGTCGGAGGC	CTGCAAGTGCATCATCGTTGTTC
TGF-β	TGATACGCCTGAGTGGCTGTCT	CACAAGAGCAGTGAGCGCTGAA
IL-10	CGGGAAGACAATAACTGCACCC	CGGTTAGCAGTATGTTGTCCAGC
CD206	GTTCACCTGGAGTGATGGTTCTC	AGGACATGCCAGGGTCACCTTT
Arg1	CATTGGCTTGCGAGACGTAGAC	GCTGAAGGTCTCTTCCATCACC
CD64	ACCTGAGTCACAGCGGCATCTA	TGACACGGATGCTCTCAGCACT

### Flow cytometry

RAW264.7 and peritoneal macrophages were pretreated as described above, and the cells were washed with PBS three times and resuspended in FACS buffer (PBS, 0.1% BSA and 0.5 mM EDTA) for staining. Cells were blocked with anti-mouse CD16/CD32 antibody (mouse Fc blocker, 1:100; BD Biosciences, USA) for 20 min on ice for flow cytometry. The cells were incubated with fluorescently labelled antibodies on ice in the dark for 20 min and then washed with FACS buffer. Macrophages were identified with V450-anti-F4/80 (eBioscience, USA) and PerCP-Cyanine5.5-anti-CD11b (eBioscience). PE-anti-CD86 (eBioscience) was used as an M1 marker, APC-anti-CD206 (Biolegend, USA) served as an M2 marker, and PE-anti-CD64 (Biolegend) was used to recognize FcγR. For analysis of intracellular molecules, the cells were permeabilized by a Fix & Perm Kit (Multisciences (Lianke) Biotech Hangzhou, China) and stained with APC-anti-CD206 intracellularly. Finally, the cells were analysed using a BD FACS Canto flow cytometer (BD Biosciences). Data were further analysed using FlowJo software (Ashland, OR, USA) [47, 48].

### Assay of nitric oxide (NO)

Based on the Griess reaction, the enriched nitrite in the medium was used as an indicator of nitric oxide (NO) production [49]. Briefly, RAW264.7 macrophages were divided into three groups: one group of macrophages was cultured with 20 µg/mL rTsDPP1, AW ESA, TRX and IL-4, and 200 ng/mL LPS alone at 37 °C for 24 h; another group was first incubated with LPS for 24 h, then cultured with rTsDPP1, AW ESA and IL-4 for 24 h; the third group was preincubated with LPS for 24 h, then cultured with the STAT6 inhibitor (AS1517499, 100 nM) or the PPARγ antagonist (GW9662, 10 µM) for 24 h [50, 51], and finally incubated with rTsDPP1, TRX, AW ESA and IL-4 for 24 h. Culture supernatant was collected and successively mixed with Griess Reagent I and II (Beyotime, Shanghai, China). After incubation at room temperature for 5 min, the absorbance of the solution at 540 nm was measured using a microplate reader. The standard curve was drawn according to the different concentrations of NaNO_2_.

### Antibody-dependent cell-mediated cytotoxicity assay

Peritoneal macrophages from normal BALB/c mice were collected as previously described [52]. The macrophages were incubated with various proteins (rTsDPP1, AW ESA, TRX, LPS and IL-4) for 24 h. The cells were washed with PBS and adjusted to 2 × 10^5^ cells in a 96-well plate with RPMI-1640 medium. Fifty newborn larvae (NBL) were added to the medium containing *T. spiralis-*infected murine serum (1:100 dilutions) and incubated at 37 °C for 72 h. In addition, the serum used in the ADCC assay was pretreated at 56 °C for 30 min to inactivate the complement component, and normal heath mouse serum was used as a negative control. To further identify the mechanisms by which rTsDPP1 inhibits CD64 expression and reduces the cytotoxicity of rTsDPP1-incubated macrophages, macrophages pretreated with a STAT6 inhibitor (AS1517499, 100 nM) or a PPARγ antagonist (GW9662, 10 µM) for 24 h were also incubated with various proteins. After the NBL were cultured with various groups of macrophages and infection serum, larval viability was assessed based on larval morphology and activity. The living NBL were active and mobile, whereas the dead NBL were inactive and straight. Cytotoxicity was defined as the percentage of dead NBL to the number of total larvae observed in each assay [53, 54].

### Statistical analysis

Statistical analysis was performed using GraphPad Prism V.9.5 (GraphPad Software Inc., USA), and data are shown as the mean ± standard error of the mean (SEM). Comparisons between two groups at multiple time points were conducted by two-way analysis of variance (ANOVA) with Sidak’s multiple comparisons test. An unpaired Student’s *t* test was used to determine the significant differences between two groups. Comparisons of more than two groups were performed using one-way ANOVA for multiple comparisons. *P* < 0.05 was defined as statistically significant.

## Results

### The effect of rTsDPP1 on the cell viability of RAW264.7 macrophages

**Figure 1:**
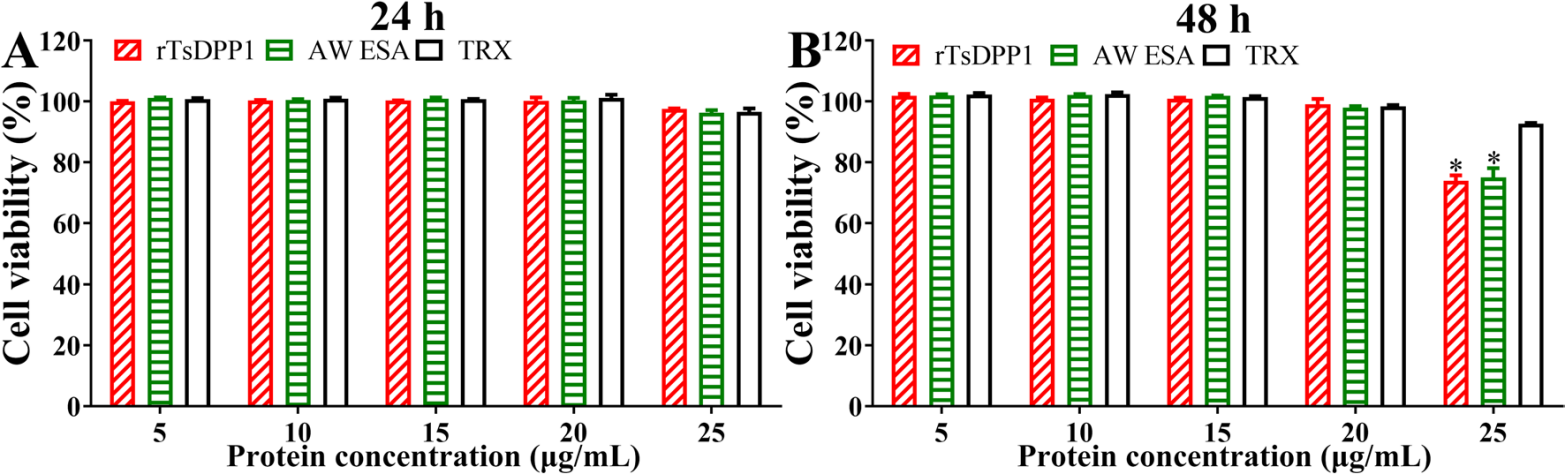
**Effect of rTsDPP1 on Raw264.7 macrophage viability assayed by CCK-8.** rTsDPP1 (5, 10, 15, 20 and 25 µg/mL) was coincubated with macrophages for 24 and 48 h to assess the effects of rTsDPP1 on cell viability. The OD_450_ values were measured by SpectraMax i3X. The OD_450_ value served as the cell proliferation index. The data were collected from 3 independent experiments and expressed as the mean ± SEM. *P < 0.05 compared to the PBS group.

After RAW264.7 macrophages were incubated with different concentrations of rTsDPP1 (5, 10, 15, 20 and 25 µg/mL) for 24 and 48 h, cell viability was evaluated by a CCK-8 assay. The results showed that 5–25 µg/mL TsDPP1 had no obvious effects on cell viability at 24 h (*F* = 0.05369, *P >* 0.05), but 25 µg/mL rTsDPP1 or AW ESA obviously decreased cell viability at 48 h compared to the PBS group (*t*_*rTsDPP1*_ = 6.028, *P <* 0.05; *t*_*AW ESA*_ = 10.551, *P <* 0.01) (Figure 1). Therefore, 20 µg/mL rTsDPP1 was used in the following experiments.

### rTsDPP1 binding to RAW264.7 macrophage membranes

**Figure 2:**
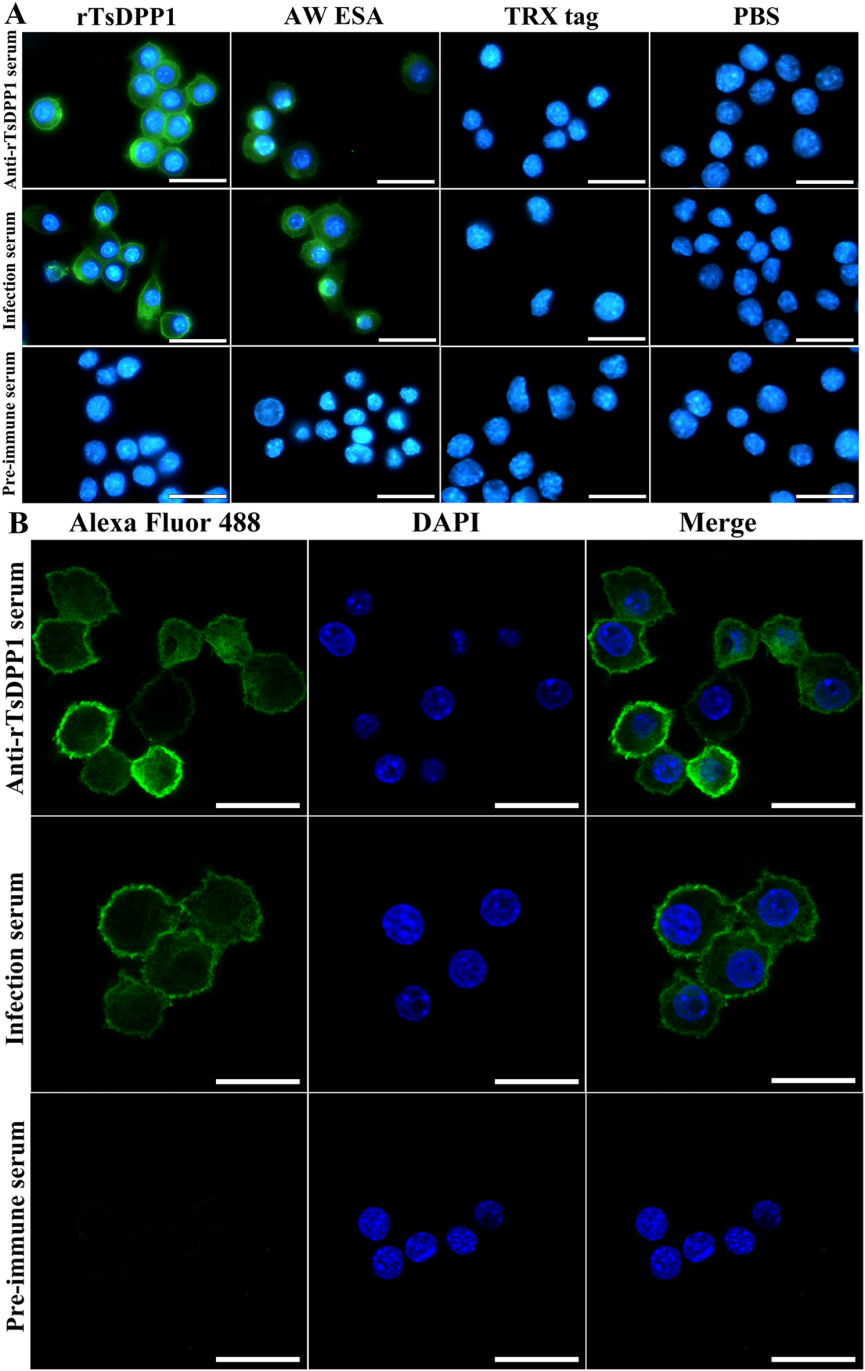
**IFT and confocal microscopy of rTsDPP1 binding to RAW264.7 macrophages.** RAW264.7 macrophages were cultured with diverse proteins at 37 °C for 2 h. Different sera (1:10; anti-rTsDPP1 serum, infection serum and preimmune serum) served as primary antibodies, and Alexa Fluor 488-conjugated anti-mouse IgG (1:100) was used as the secondary antibody. Cell nuclei were stained with DAPI. **A** Specific binding of rTsDPP1 to macrophages was observed under a fluorescence microscope. **B** The binding site of rTsDPP1 with macrophages was localized on the cell membrane by confocal microscopy. Scale bars: 25 μm.

To investigate the binding between rTsDPP1 and macrophages, IFT and confocal microscopy were performed. The results showed that after incubation with rTsDPP1, bright green fluorescence on the surface of macrophages was detected by anti-rTsDPP1 serum and infection serum (Figure 2A). Confocal microscopy revealed that the binding site of rTsDPP1 and macrophages was localized on the cell membrane (Figure 2B). The results indicated that rTsDPP1 had the ability to bind to macrophages.

### rTsDPP1 promotes macrophage M2 polarization via the STAT6/PPARγ pathway

**Figure 3:**
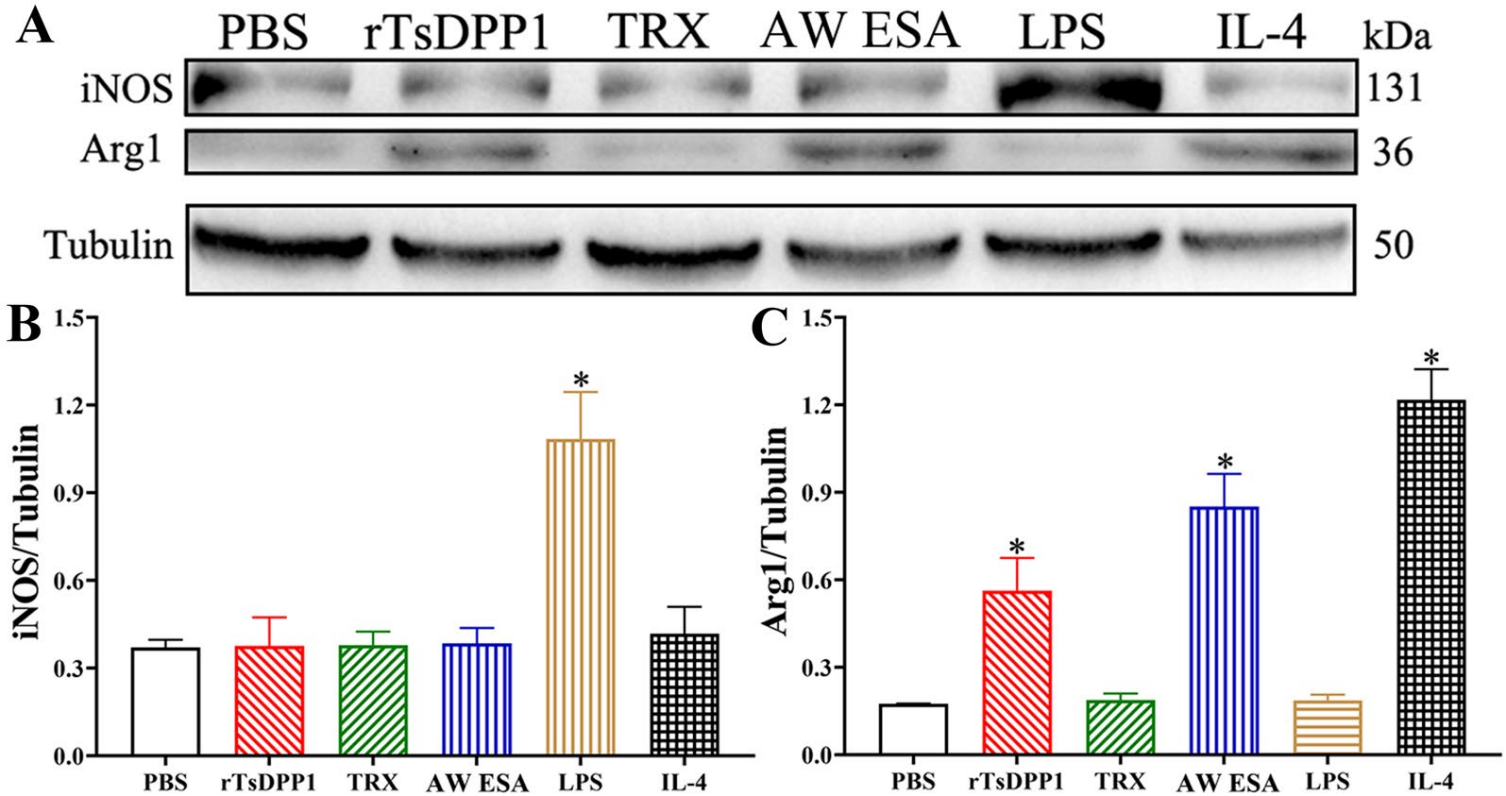
**Expression level of M1/M2 marker proteins in RAW264.7 macrophages stimulated by rTsDPP1.** The expression of iNOS and Arg1 in RAW264.7 macrophages was analysed by Western blotting after the cells were incubated with various stimulators for 24 h. **A** Expression of iNOS and Arg1 in various groups of cells. Tubulin served as the internal control. **B** The intensity of iNOS protein signal relative to the intensity of tubulin control. **C** The intensity of the Arg1 protein signal relative to the intensity of the tubulin control. *P < 0.05 compared to the PBS group.

**Figure 4:**
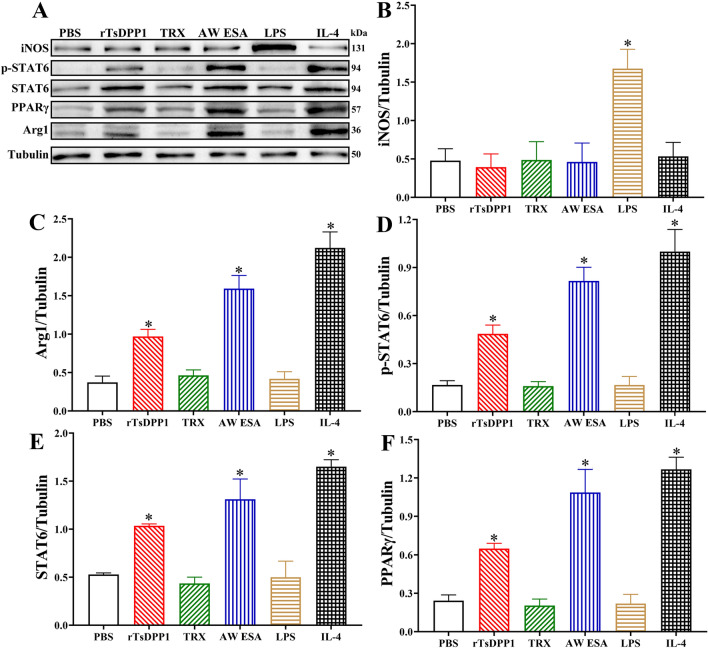
**Expression levels of M1/M2 marker proteins in murine peritoneal macrophages.** Peritoneal macrophages were collected from normal BALB/C mice and treated with various stimulating factors. **A** Western blotting of the expression levels of iNOS, p-STAT6, STAT6, PPARγ and Arg1 in various groups of treated macrophages. Tubulin served as the internal control. **B**–**F** The protein signal intensity of iNOS, Arg1, p-STAT6, STAT6, and PPARγ relative to the intensity of tubulin. *P < 0.05 compared to the PBS group.

To evaluate the roles of rTsDPP1 in macrophage polarization, the murine monocyte/macrophage line RAW264.7 and murine peritoneal macrophages were used for the in vitro stimulation of M1 and M2 phenotypes. Western blotting showed that after RAW264.7 macrophages were stimulated with 20 µg/mL rTsDPP1 or AW ESA at 37 °C for 2 h, the expression level of iNOS (M1) was not evidently changed compared to that in the PBS group (*F* = _9.__895, *P*rTsDPP1_ *>* 0.05, *P*_*ESA*_ *>* 0.05), but the protein expression level of Arg1 (M2) was obviously increased (*F* = 30.44, *P*_rTsDPP1_ *<* 0.05, *P*_ESA_ *<* 0.001) (Figures 3A–C). To further study the mechanism by which rTsDPP1 induces macrophage polarization, murine peritoneal macrophages were also stimulated. The results showed that the expression level of iNOS (M1) was not statistically changed compared to that in the PBS group (*F* = 5.479, *P*_rTsDPP1_ *>* 0.05, *P*_ESA_ *>* 0.05), but the Arg1 expression level in the rTsDPP1 and AW ESA groups was significantly higher than that in the PBS group (*F* = 31.22, *P*_rTsDPP1_ *<* 0.05, *P*_AW ESA_ *<* 0.001) (Figures 4A–C), which was consistent with the findings in RAW264.7 macrophages. Moreover, the protein expression of p-STAT6, STAT6 and PPARγ in the rTsDPP1-treated group was evidently increased compared to that in the PBS group (*P <* 0.05) (Figures 4D–F). The results suggested that rTsDPP1 elicited macrophage M2 polarization in vitro, as demonstrated by the expression level of Arg1 (M2 marker) being clearly increased via the activation of the STAT6/PPARγ pathway.

### Upregulation of M2-related genes in rTsDPP1-treated macrophages

**Figure 5:**
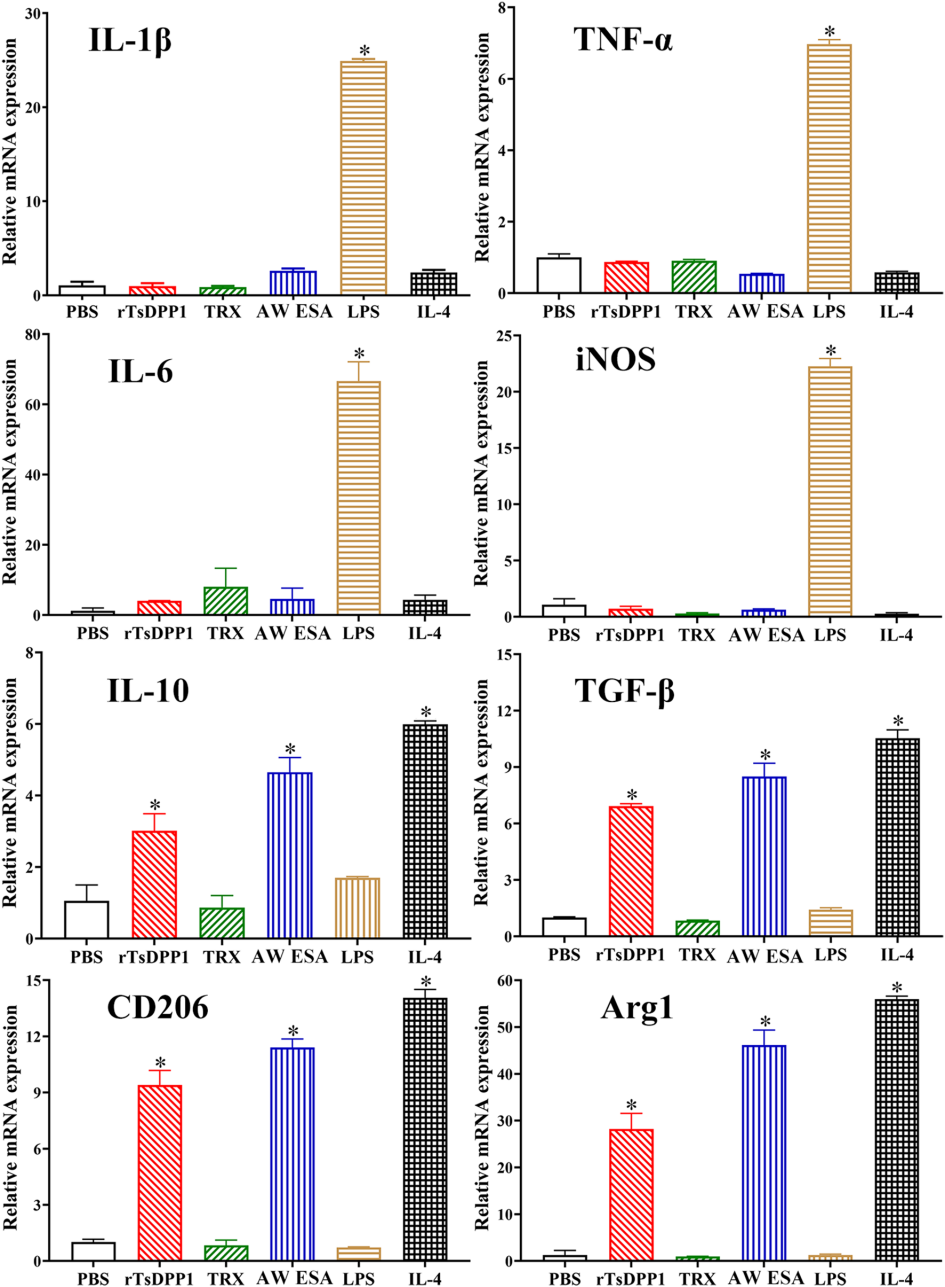
**The mRNA expression levels of M1/M2-related genes in RAW264.7 macrophages stimulated by rTsDPP1.** The mRNA expression of M1/M2-related genes in RAW264.7 macrophages treated with various proteins was analysed by qPCR. The M1 macrophage-related genes are IL-1β, TNF-α, IL-6 and iNOS. The M2 macrophage-related genes are IL-10, TGF-β, CD206 and Arg1. The mRNA expression levels of the genes were calculated with the Ct^(2−ΔΔCt)^ method. GAPDH was utilized as an internal control. *P < 0.05 compared to the PBS group.

**Figure 6:**
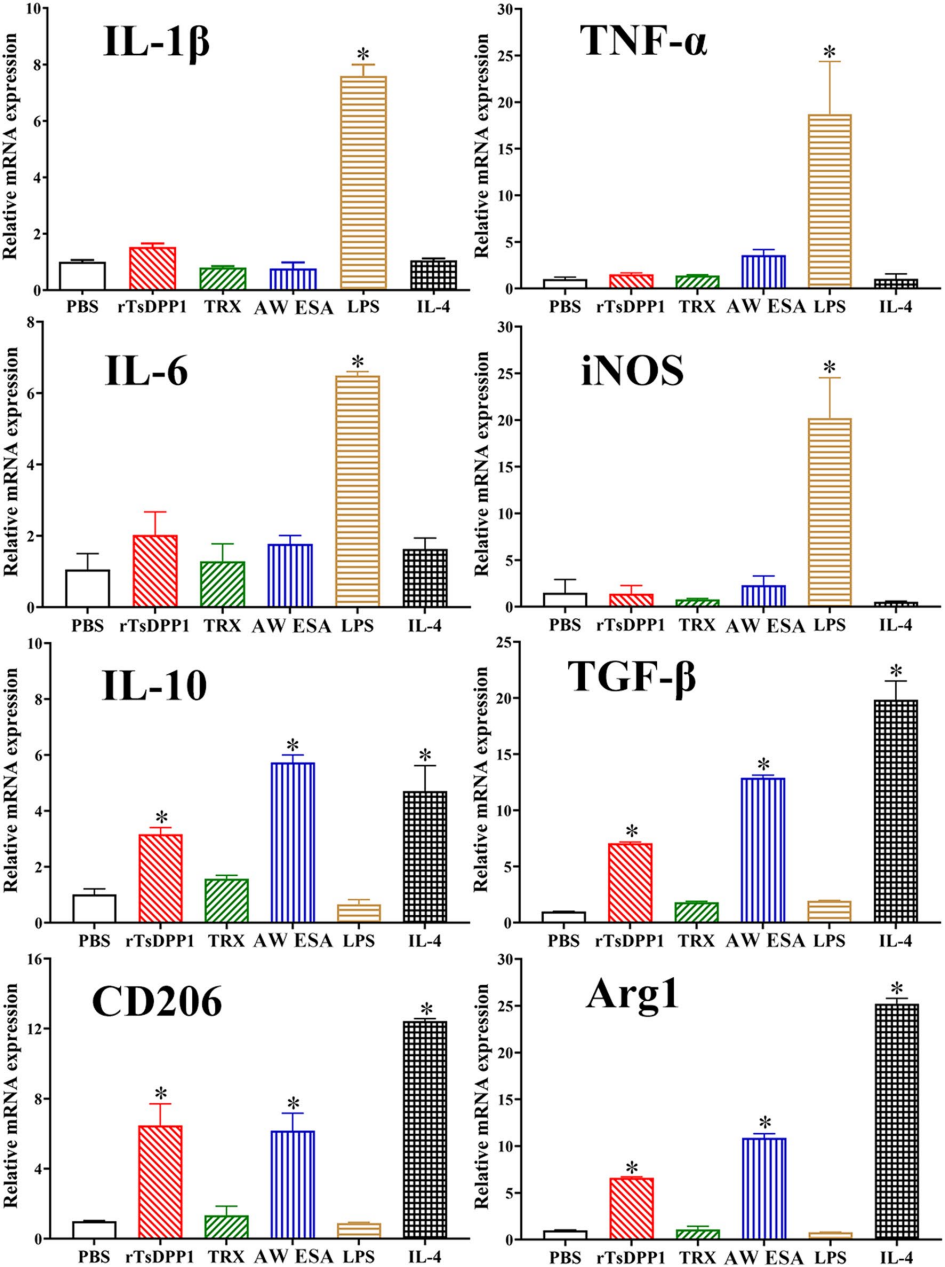
**The mRNA expression level of M1/M2-related genes in mouse peritoneal macrophages stimulated by rTsDPP1.** The mRNA expression of M1/M2-related genes in peritoneal macrophages treated with various proteins was analysed by qPCR. The mRNA expression levels of M1 macrophage-related genes (IL-1β, TNF-α, IL-6 and iNOS) and M2 macrophage-related genes (IL-10, TGF-β, CD206 and Arg1) were assayed by qPCR and calculated with the Ct ^(2−ΔΔCt)^ method. GAPDH was utilized as an internal control. *P < 0.05 compared to the PBS group.

To investigate the role of rTsDPP1 in macrophage polarization, RAW264.7 and peritoneal macrophages were treated with various proteins, and the mRNA expression levels of M1/M2-related genes were analysed by qPCR. The results revealed that in rTsDPP1-treated RAW264.7 macrophages, the mRNA expression of M1 genes (IL-1β, TNF-α, IL-6 and iNOS) was not obviously changed compared to that in the PBS group (*F*_IL−1β_ = 3525, *F*_TNF−α_ = 4099, *F*_IL−6_ = 165.8, *F*_iNOS_ = 1725, *P >* 0.05), but rTsDPP1 evidently upregulated the mRNA expression of M2 genes (IL-10, TGF-β, CD206 and Arg1) compared to that in the PBS group (*F*_IL−10_ = 109.4, *F*_TGF−β_ = 453.0, *F*_CD206_ = 605.9, *F*_Arg1_ = 482.7, *P <* 0.0001) (Figure 5). Furthermore, after murine peritoneal macrophages were treated with rTsDPP1, compared to the PBS group, the mRNA expression of M1 genes was not obviously changed (*F*_IL−1β_ = 569.4, *F*_TNF−α_ = 27.14, *F*_IL−6_ = 74.22, *F*_iNOS_ = 48.22, *P >* 0.05), but the mRNA expression of M2 genes was significantly increased (*F*_IL−10_ = 75.52, *F*_TGF−β_ = 359.7, *F*_CD206_ = 134.8, *F*_Arg1_ = 2489, *P* < 0.0001) (Figure 6). The results demonstrated that rTsDPP1 induced the activation of M2 phenotypic macrophages (Arg-1 and CD206) and evidently increased the mRNA expression levels of anti-inflammatory cytokines (IL-10 and TGF-β) in M2-polarized macrophages.

### rTsDPP1 induced high expression of the M2 marker CD206 in macrophages

**Figure 7:**
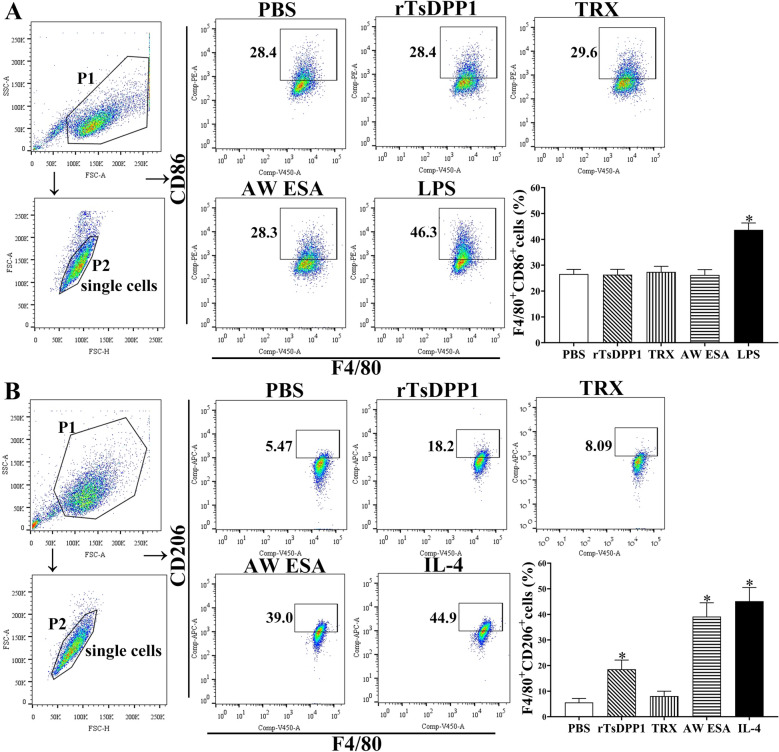
**Expression of M1/M2 markers in RAW264.7 macrophages by flow cytometry.** The expression of CD86 (M1 marker) and CD206 (M2 marker) was analysed in RAW264.7 macrophages stimulated with various stimulators. The single cell was labelled with forwards scatter-area (FSC-A) and forwards scatter-height (FSC-H). **A** The black box displays the M1 cells (F4/80^+^ and CD86^+^), and the percentage of CD86-positive cells is shown in the bar graph. **B** The black box displays the M2 cells (F4/80^+^ and CD206^+^), and the percentage of CD206-positive cells is shown in the bar graph. *P < 0.05 compared to the PBS group.

**Figure 8:**
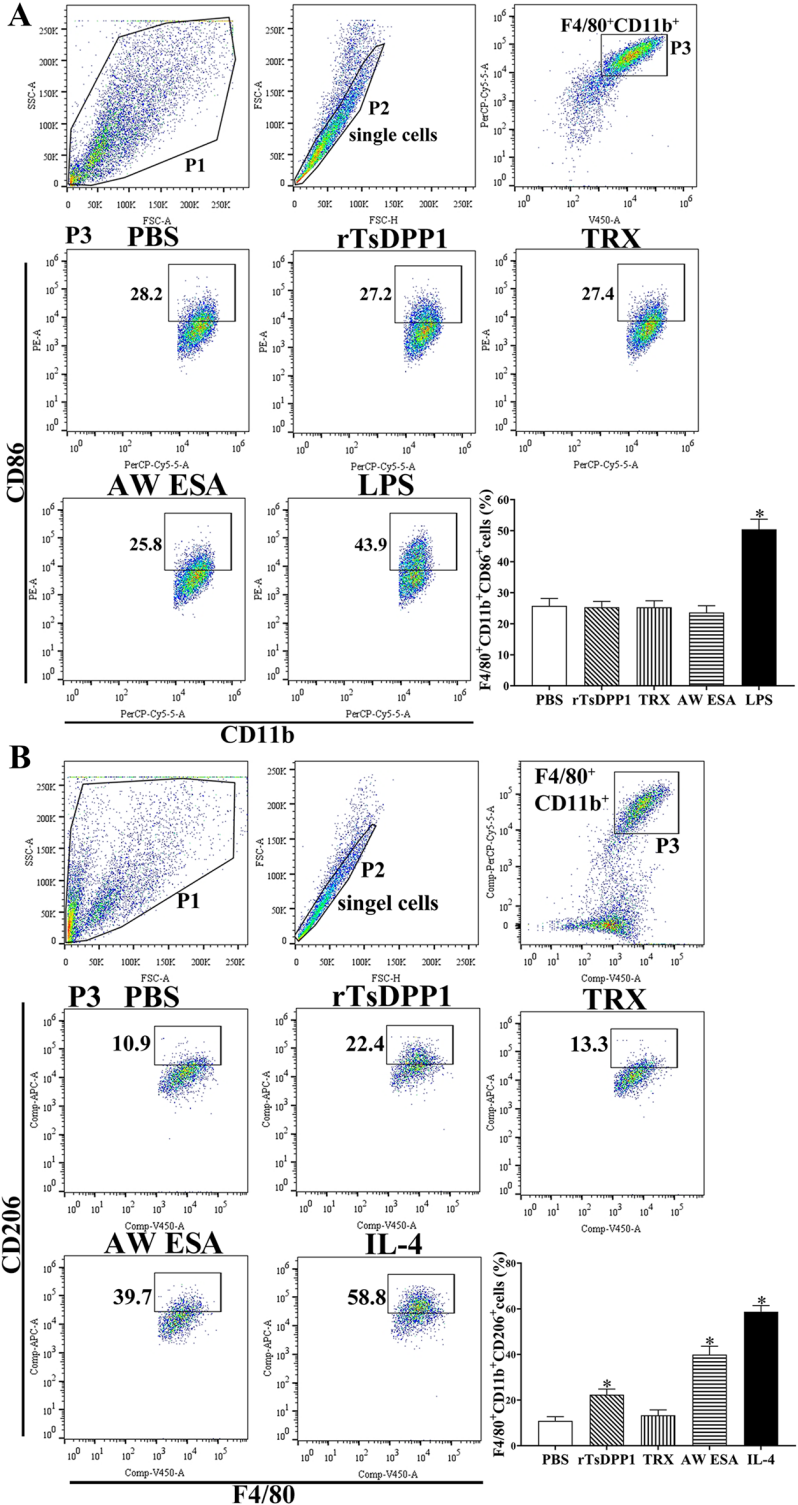
**Expression of M1/M2 markers in murine peritoneal macrophages by flow cytometry.** The cell population was analysed in the P1 gate, and a single cell in P2 was circled using forwards scatter-area (FSC-A) and forwards scatter-height (FSC-H). Macrophages were identified using F4/80 and CD11b in the P3. The expression of CD86 (M1 marker) and CD206 (M2 marker) was analysed after stimulation with various stimulators. **A** The black box displays the M1 cells (F4/80^+^, CD11b^+^ and CD86^+^), and the percentage of CD86-positive cells is shown in the bar graph. **B** The black box displays the M2 cells (F4/80^+^, CD11b^+^ and CD206^+^), and the percentage of CD206-positive cells is shown in the bar graph. *P < 0.05 compared to the PBS group.

Flow cytometry was conducted to further investigate rTsDPP1-induced macrophage polarization. RAW264.7 macrophages were gated by F4/80, and CD86 and CD206 were used as M1 and M2 markers, respectively. The results showed that compared to that in the PBS group, the M1 marker (CD86) expression level was not changed in cells treated with rTsDPP1 (*F* = 37.17, *P* = 0.9998) (Figure 7A), but the M2 marker (CD206) expression level was obviously increased (*F* = 63.89, *P <* 0.01) (Figure 7B). Similarly, after murine peritoneal macrophages were stimulated with rTsDPP1 and AW ESA, compared to the PBS group, no significant change in M1 marker (F4/80 + CD11b + CD86 + cells) expression level was observed (*F* = 67.16, *P* ˃ 0.05) (Figure 8A); however, the macrophages showed a high expression of F4/80 + CD11b + CD206 + cells (*F* = 168.6, *P <* 0.01) (Figure 8B), demonstrating that rTsDPP1 promoted macrophage polarization towards the M2 type.

### STAT6 inhibitor and PPARγ antagonist abrogated rTsDPP1-induced M2 polarization

**Figure 9:**
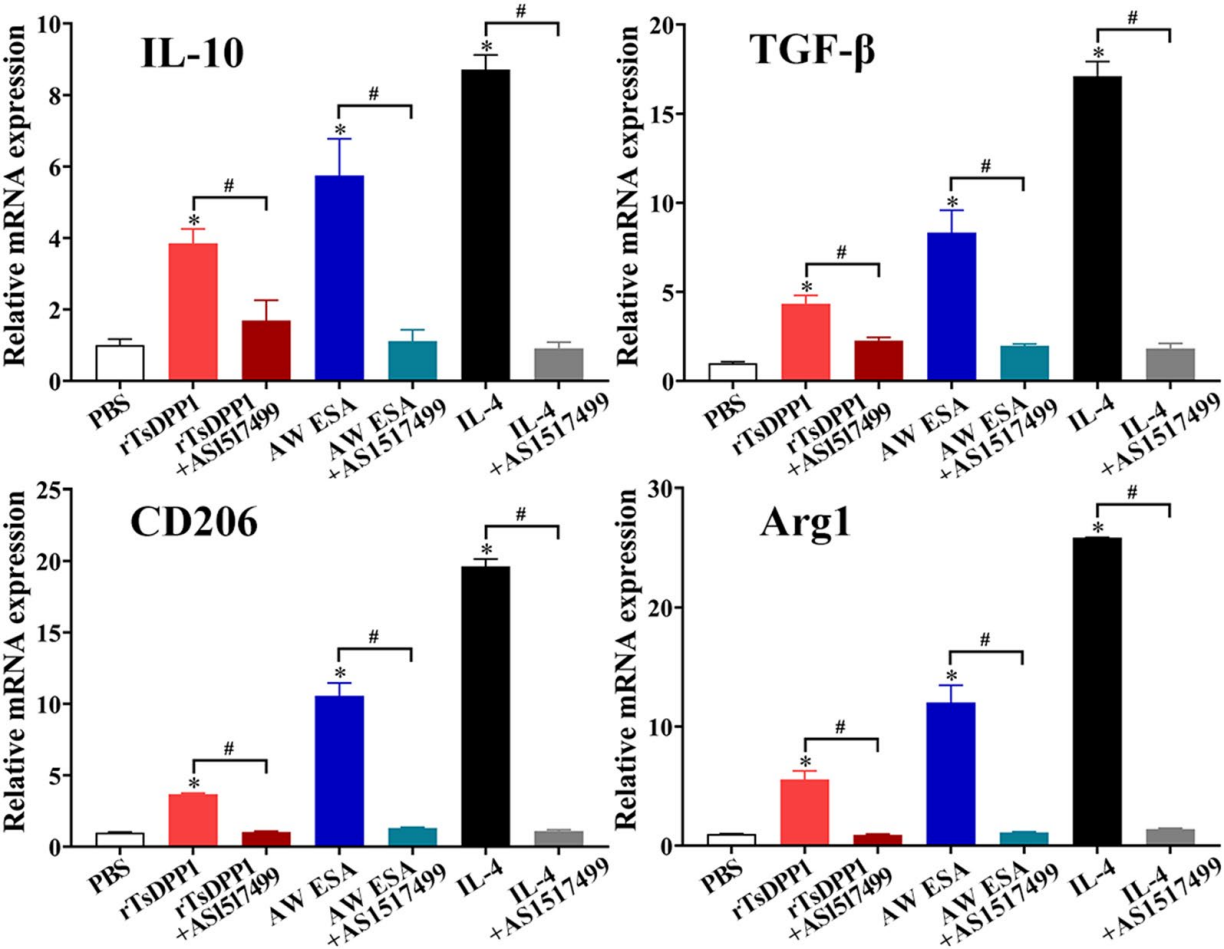
**STAT6 inhibitor abrogated the rTsDPP1 induced-M2 polarization by qPCR.** Mouse peritoneal macrophages were pretreated with a STAT6 inhibitor (AS1517499) and cultured with rTsDPP1 at 37 °C and 5% CO_2_ for 24 h. Total RNA was extracted and reverse transcribed to cDNA for qPCR. The relative mRNA expression of M2 macrophage genes (IL-10, TGF-β, CD206 and Arg1) was calculated with the Ct ^(2−ΔΔCt)^ method. GAPDH was utilized as an internal control. *P < 0.05 compared to the PBS group, ^#^*P* < 0.05 between two groups.

**Figure 10:**
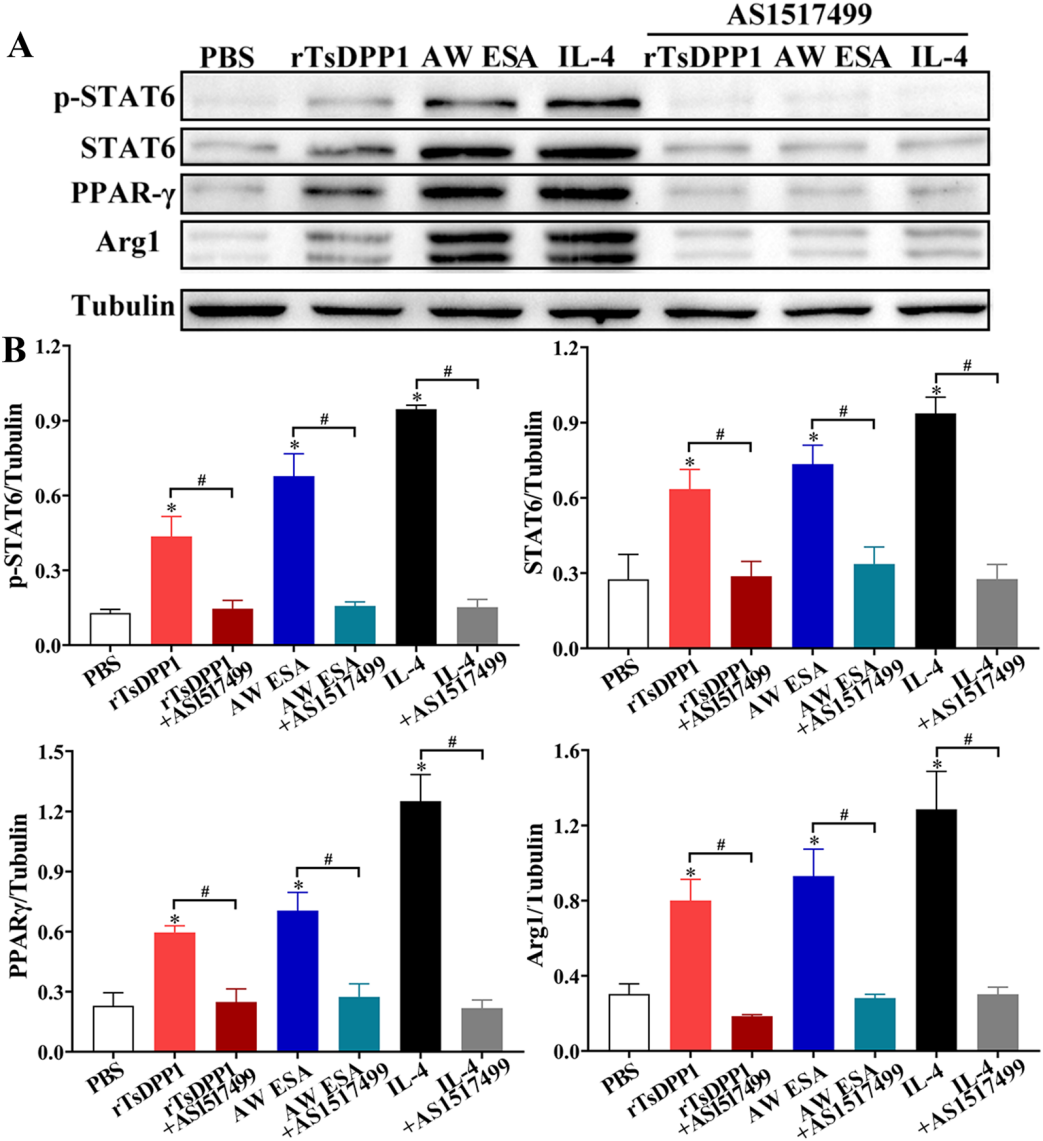
**Western blotting analysis of STAT6 inhibitor abrogating rTsDPP1-induced M2 polarization.** Mouse peritoneal macrophages were pretreated with a STAT6 inhibitor (AS1517499) and cultured with rTsDPP1 at 37 °C and 5% CO_2_ for 24 h. **A** Western blotting of the expression of p-STAT6, STAT6, PPARγ and Arg1 in macrophages pretreated with a STAT6 inhibitor. Tubulin served as the internal control. **B** The intensity of p-STAT6, STAT6, PPARγ and Arg1 protein relative to the tubulin intensity. *P < 0.05 compared to the PBS group, ^#^*P* < 0.05 between two groups.

As described above in the subsection “rTsDPP1 promotes macrophage M2 polarization via the STAT6/PPARγ pathway”, rTsDPP1 induced macrophage M2 polarization through the activation of the STAT6/PPARγ pathway. To further confirm the critical roles of STAT6 and PPARγ signals in rTsDPP1 triggering peritoneal macrophage polarization, the STAT6 inhibitor (AS1517499, 100 nM) and the PPARγ antagonist (GW9662, 10 µM) were preincubated with mouse peritoneal macrophages at 37 °C for 2 h. The qPCR results showed that the transcript levels of M2 genes (IL-10, TGF-β, CD206 and Arg1) were obviously downregulated by the STAT6 inhibitor compared to the rTsDPP1 only group (*t*_IL−10_ = 5.403, *t*_TGF−β_ = 7.213, *t*_CD206_ = 48.940, *t*_Arg1_ = 11.253, *P* < 0.01) (Figure 9). Western blotting results revealed that the rTsDPP1-upregulated expression levels of p-STAT6, STAT6, PPARγ and Arg1 were also significantly reduced and restored to the PBS group level by the STAT6 inhibitor (*t*_p−STAT6_ = 3.326, *t*_STAT6_ = 3.538, *t*_PPARγ_ = 4.753, *t*_Arg1_ = 5.395, *P* < 0.05) (Figure 10). The results suggested that the STAT6 inhibitor completely inhibited and blocked the STAT6/PPARγ pathway and the activation of macrophage M2 polarization.

**Figure 11:**
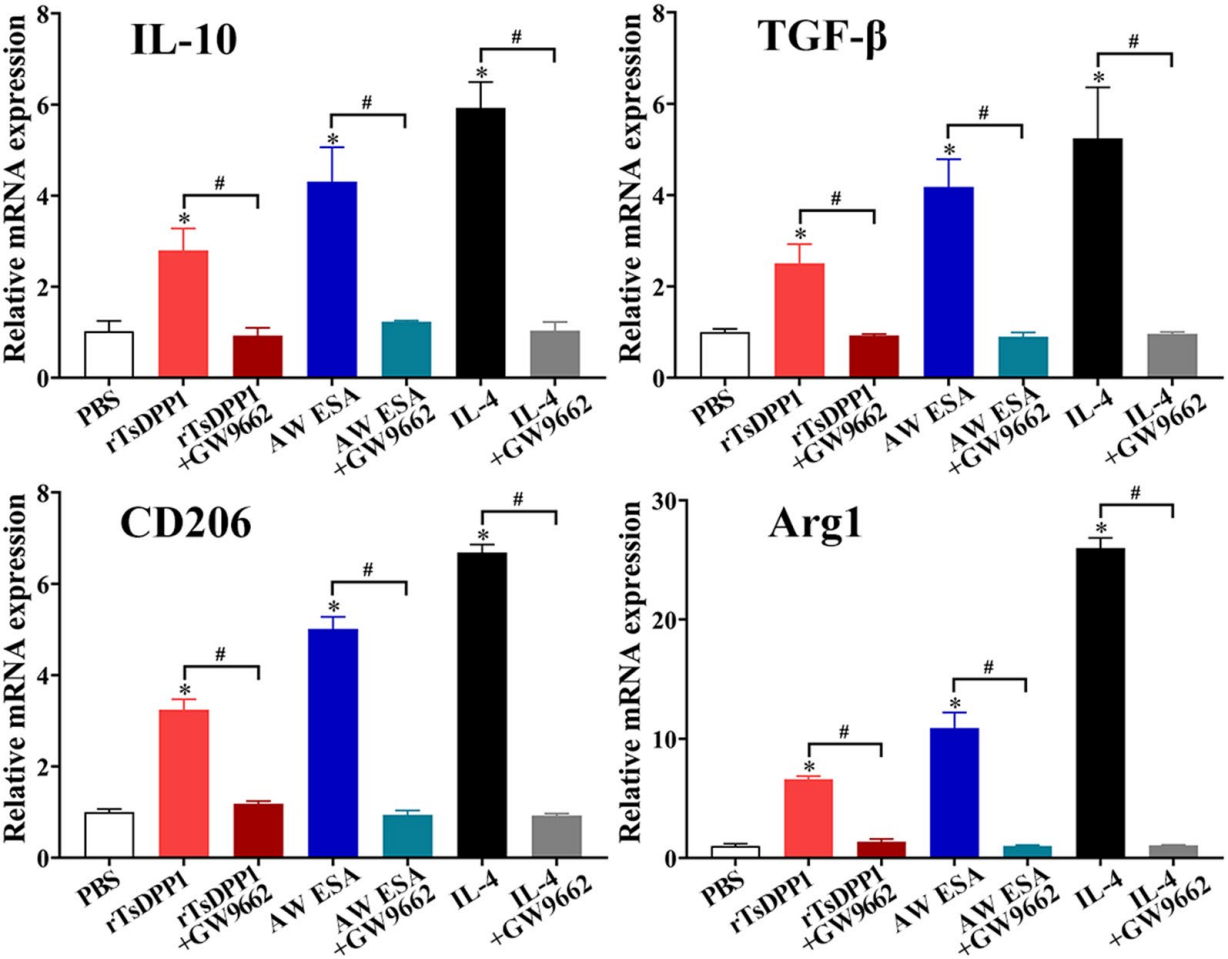
**PPARγ antagonist abrogated the rTsDPP1-induced high mRNA expression of M2 genes by qPCR.** Mouse peritoneal macrophages were pretreated with a PPARγ antagonist (GW9662) and then incubated with rTsDPP1 at 37 °C and 5% CO_2_ for 24 h. Total RNA was isolated and reverse transcribed to cDNA for qPCR. The relative mRNA expression levels of M2 genes (IL-10, TGF-β, CD206 and Arg1) were calculated with the Ct ^(2−ΔΔCt)^ method. GAPDH was used as an internal control. *P < 0.05 compared to the PBS group, ^#^*P* < 0.05 between two groups.

**Figure 12:**
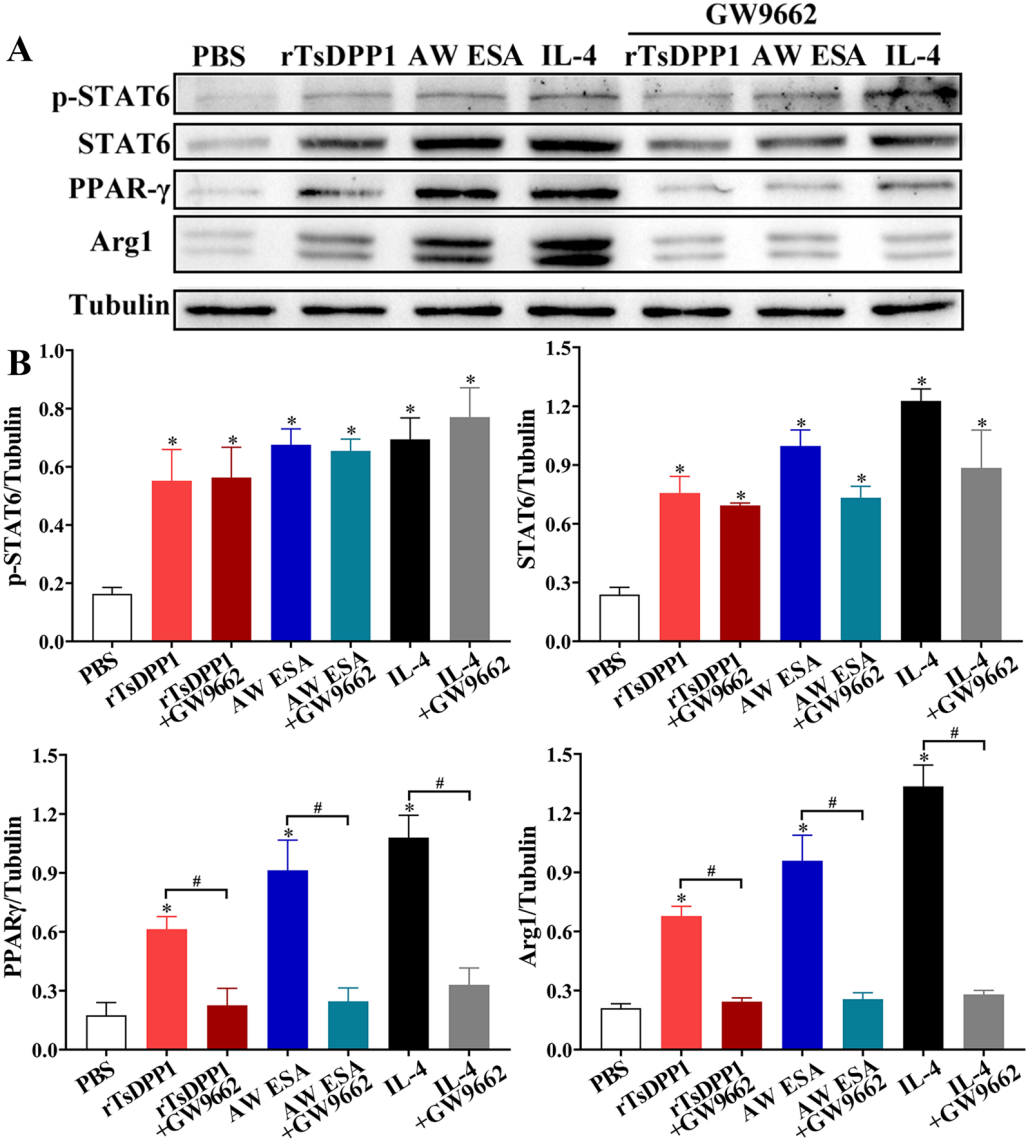
**Western blotting analysis of PPARγ antagonist abrogating the rTsDPP1-induced M2 polarization.** Mouse peritoneal macrophages were pretreated with a PPARγ antagonist (GW9662) and cultured with rTsDPP1 at 37 °C and 5% CO_2_ for 24 h. The soluble cell proteins from treated peritoneal macrophages were separated by SDS‒PAGE and analysed by Western blotting. **A** Western blotting of M2 polarization markers and pathways (p-STAT6, STAT6, PPARγ and Arg1). Tubulin served as an internal control. **B** The intensity of p-STAT6, STAT6, PPARγ and Arg1 protein relative to the intensity of tubulin. *P < 0.05 compared to the PBS group, ^#^*P* < 0.05 between two groups.

Furthermore, compared to the PBS group, the mRNA expression levels of M2 cytokines and markers (IL-10, TGF-β, CD206 and Arg1) were notably decreased after macrophages were treated with a PPARγ antagonist (GW9662) (*t*_IL−10_ = 6.334, *t*_TGF−β_ = 6.457, *t*_CD206_ = 14.836, *t*_Arg1_ = 25.872, *P* < 0.01) (Figure 11). Although the PPARγ antagonist (GW9662) had no obvious impact on the rTsDPP1-induced high expression of p-STAT6 and STAT6 (*t*_p−STAT6_ = 0.07794, *t*_Arg1_ = 0.7620, *P* ˃ 0.05), after peritoneal macrophages were treated with the PPARγ antagonist, the expression levels of PPARγ and Arg1 (M2 marker) were obviously reduced (*t*_PPARγ_ = 3.581, *t*_Arg1_ = 8.026, *P* < 0.05) (Figure 12). The results indicated that rTsDPP1 directly facilitated M2 macrophage polarization by activating the STAT6-PPARγ signalling pathway, and the facilitation role was completely abrogated by a STAT6 inhibitor and PPARγ antagonist.

### The inhibition of macrophage NO production by rTsDPP1

**Figure 13:**
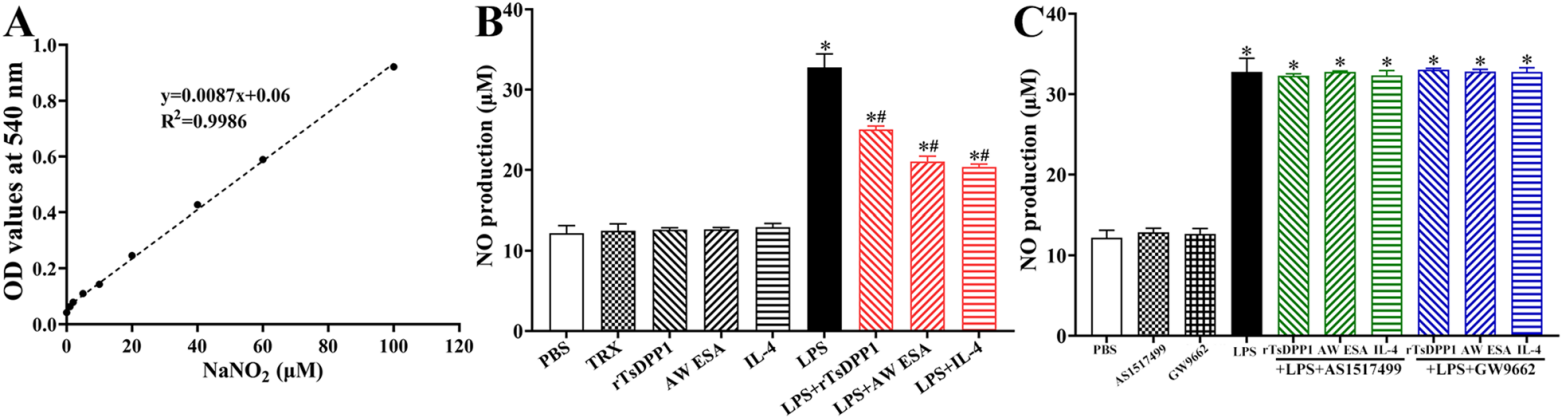
**NO production in the supernatant of cultured RAW264.7 macrophages. A** The standard curve of NO concentration. **B** Macrophages were cultured with only rTsDPP1, AW ESA, TRX, IL-4 or LPS, or after being precultured with LPS, macrophages were recultured with different proteins (rTsDPP1, AW ESA and IL-4). **C** Macrophages were preincubated with LPS for 24 h, cultured with a STAT6 inhibitor (AS1517499, 100 nM) or a PPARγ antagonist (GW9662, 10μM) for 24 h, and finally incubated with rTsDPP1, AW ESA and IL-4 for 24 h,. LPS (200 ng/mL) was used as a positive control, while IL-4 (20 ng/mL) was used as a negative control. *P < 0.05 compared to the PBS group, ^*#*^*P* < 0.05 compared to the LPS group.

To assess the effect of rTsDPP1 on NO production in RAW264.7 macrophages, a Griess reaction was performed. The standard curve of NO concentration was constructed according to the OD values at 540 nm of a series of concentrations of NaNO_2_ (Figure 13A). The results showed that NO production in LPS-treated macrophages was obviously increased compared to that in the PBS group (*F* = 259.2, *P* < 0.0001), but NO production in macrophages treated only with rTsDPP1, AW ESA, TRX or IL-4 was not evidently changed relative to that in the PBS group (*F* = 259.2, *P* > 0.05). Moreover, after incubation with rTsDPP1, AW ESA and IL-4, NO production in LPS-treated macrophages was also significantly decreased compared to that in the LPS-only group (*F* = 259.2, *P <* 0.0001) (Figure 13B). However, both the STAT6 inhibitor and PPARγ antagonist also abrogated the function of rTsDPP1 in reducing LPS-induced NO production (*F* = 537.8, *P* > 0.05); namely, the inhibitor and antagonist also increased and restored NO production in LPS + rTsDPP1-treated macrophages (Figure 13C). These results suggest that rTsDPP1 suppressed LPS-induced NO production in macrophages through the STAT6/PPARγ pathway.

### rTsDPP1 suppressed the mRNA expression of FcγR I (CD64) and cytotoxicity of macrophages via the STAT6/PPARγ pathway

**Figure 14:**
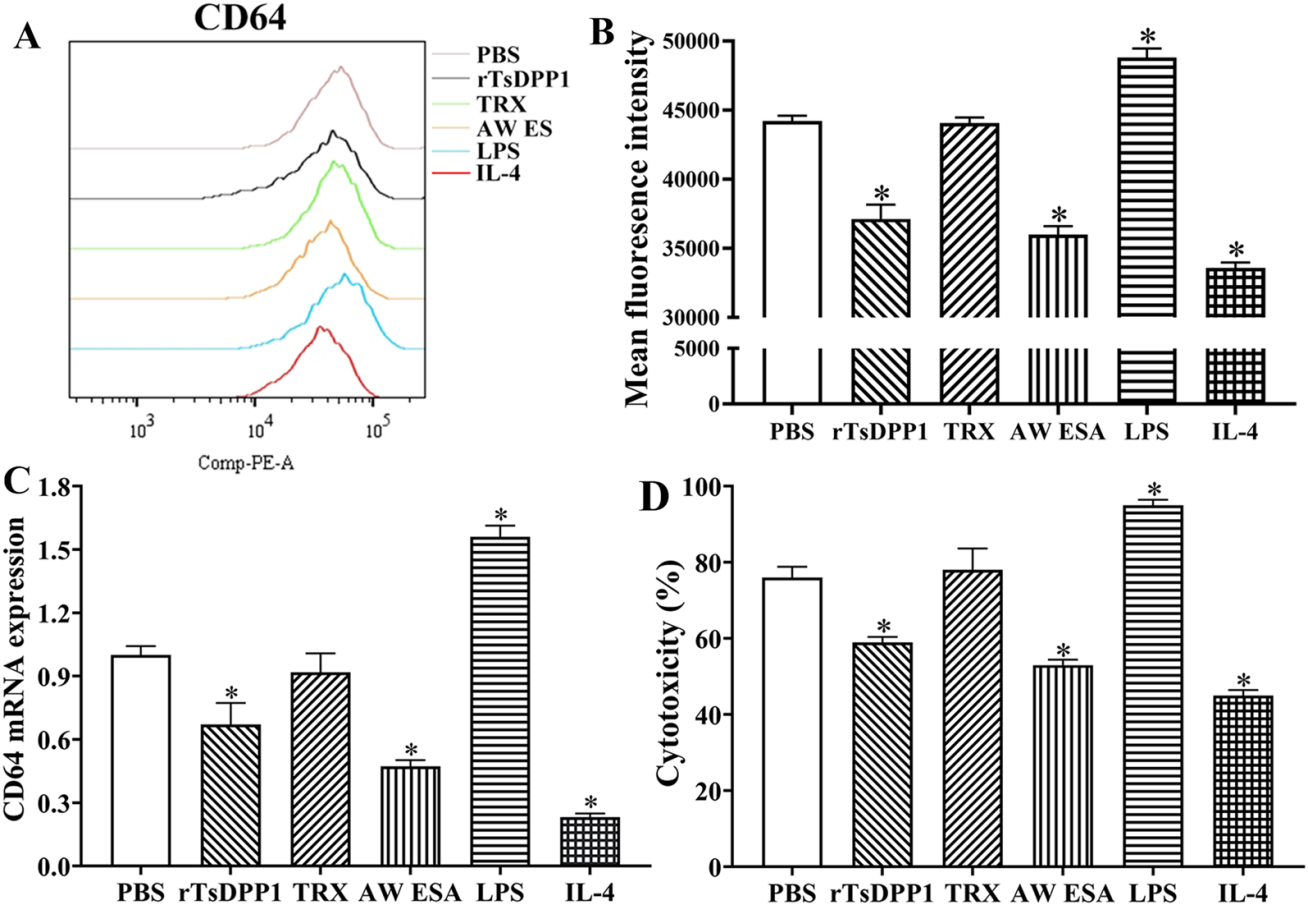
**rTsDPP1 downregulated the expression of FcγR I (CD64) in macrophages and reduced the ability of ADCC to kill larvae. A** CD64 expression in rTsDPP1-treated macrophages was measured by flow cytometry. **B** The mean fluorescence intensity of CD64^+^ macrophages. **C** qPCR assay of CD64 expression in rTsDPP1-treated macrophages. **D** Killing effect of anti-*Trichinella* antibody-mediated ADCC on NBL. rTsDPP1-treated macrophages and *T. spiralis*-infected mouse serum (1:100 dilutions) were used in the ADCC assay. The cytotoxicity was ascertained as the percentage of dead larvae to the total larvae observed in each test. *P < 0.05 compared to the PBS group.

To identify the effect of rTsDPP1 on the expression of FcγR I (CD64) on macrophages, macrophages cultured with rTsDPP1 were analysed by flow cytometry. The results showed that the mean fluorescence intensity of CD64^+^ macrophages was decreased by 15.72% compared to that in the PBS group (*F* = 265.1, *P* < 0.0001) (Figures 14A and B), indicating that rTsDPP1 significantly suppressed CD64 expression on the surface of macrophages. Furthermore, qPCR results demonstrated that after macrophages were incubated with rTsDPP1, CD64 mRNA expression on macrophages was reduced by 32.90% compared to that in the PBS group (*F* = 161.0, *P* < 0.01) (Figure 14C). The results suggested that the binding capacity of FcγR I on macrophages with anti-*Trichinella* IgG antibodies was evidently decreased due to the reduction in CD64 expression levels on macrophages treated with rTsDPP1. Subsequently, the ability of macrophages to kill NBL through ADCC was obviously reduced, and the cytotoxicity (larval death rate) of rTsDPP1-treated macrophages was decreased by 22.37% compared to that of the PBS group (*F* = 86.37, *P* < 0.01) (Figure 14D). This result was consistent with that of rTsDPP1 activating macrophage M2 polarization, which decreased the expression of killing molecules (NO) and anti-inflammatory cytokines (IL-10 and TGF-β). The results suggested that rTsDPP1-activated macrophage M2 polarization and decreased FcγR I expression might be beneficial to the survival of *T. spiralis* nematodes and mediate their immune evasion in hosts.

**Figure 15:**
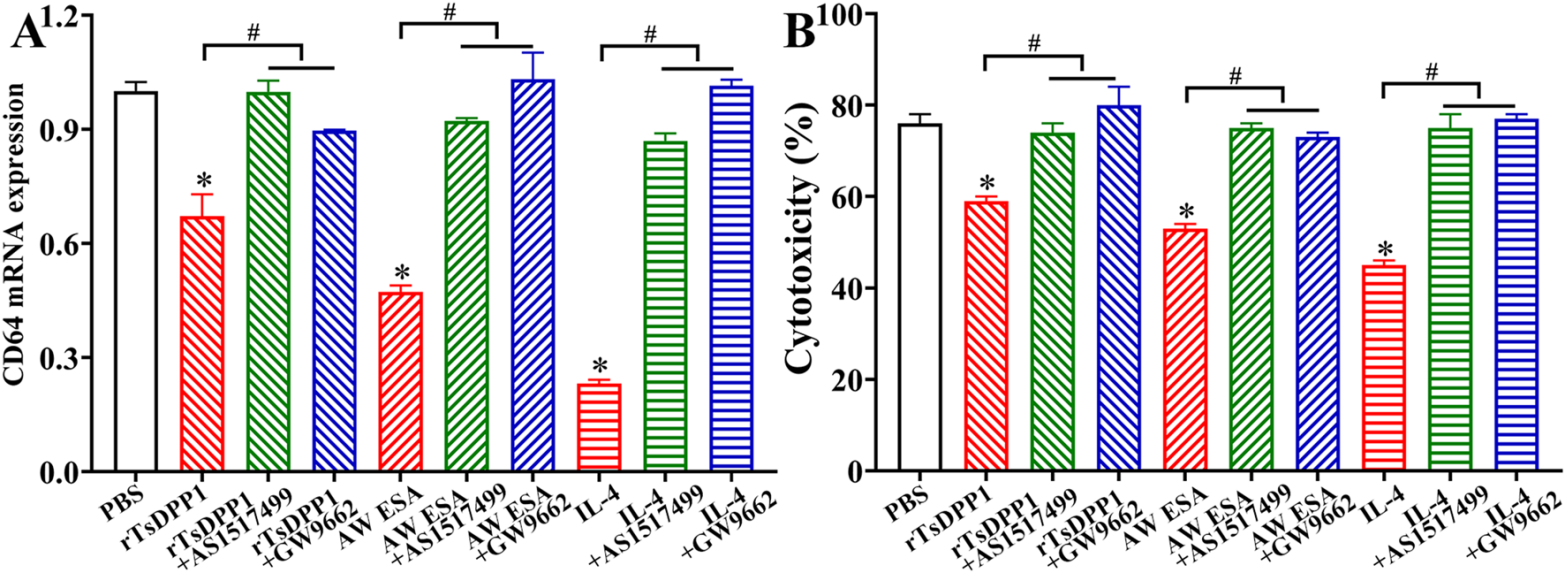
** A STAT6 inhibitor (AS1517499) and PPARγ antagonist (GW9662) restored the expression of rTsDPP1-suppressed CD64 and the cytotoxicity of macrophages. A** After macrophages were cultured with AS1517499 or GW9662, the mRNA expression of CD64 was assessed by qPCR. **B** ADCC killing effects on *T. spiralis* NBL. Macrophages preincubated with a STAT6 inhibitor (AS1517499, 100 nM) or a PPARγ antagonist (GW9662, 10 µM) were again incubated with various proteins for 24 h. Cytotoxicity was calculated as the percent of dead NBL to total larvae observed in each assay. *P < 0.001 compared to the PBS group, #* P* < 0.001 between two groups.

**Figure 16:**
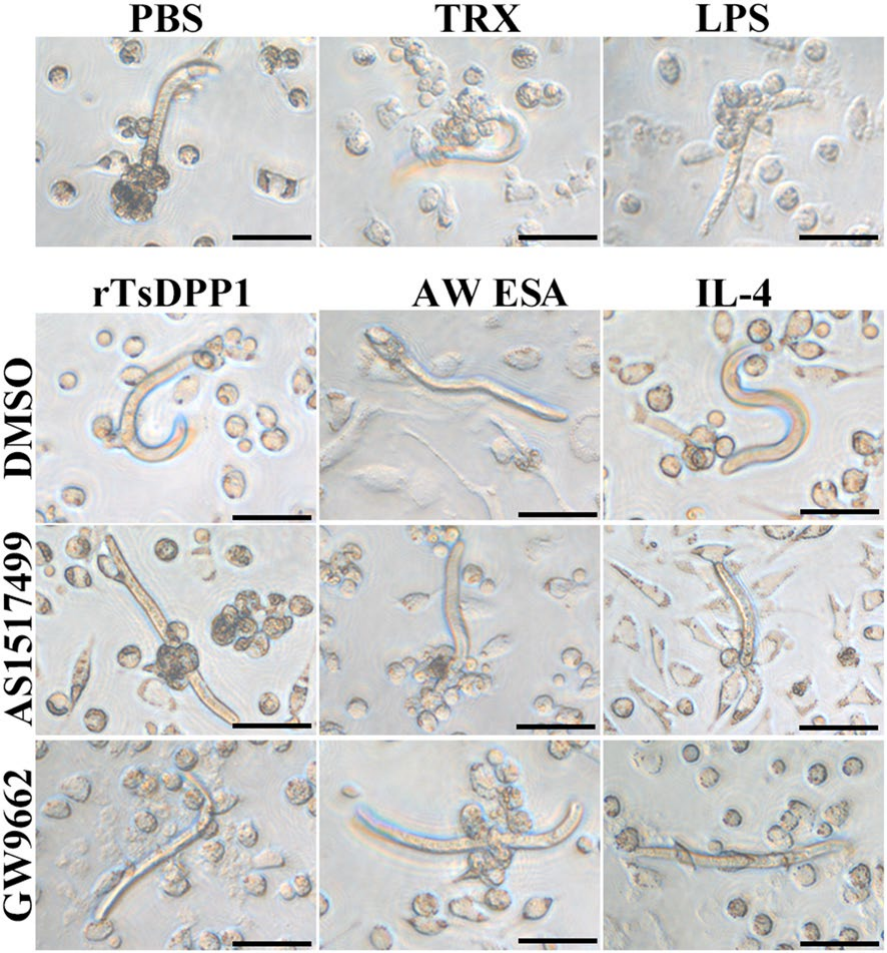
**The killing effect of ADCC on NBL by rTsDPP1-treated peritoneal macrophages.** Peritoneal macrophages were collected from normal BALB/c mice, pretreated with a STAT6 inhibitor (AS1517499, 100 nM) or a PPARγ antagonist (GW9662, 10 µM) for 24 h, incubated with rTsDPP1 for 24 h, and finally incubated with NBL (50 larvae/well) for 72 h. The ADCC assay was performed using infection serum (1:100) and observed by microscopy. Scale bars: 25 μm.

To further identify the mechanisms by which rTsDPP1 inhibits CD64 expression and reduces the cytotoxicity of rTsDPP1-incubated macrophages, a STAT6 inhibitor (AS1517499) and PPARγ antagonist (GW9662) were also used to treat macrophages. The qPCR results showed that both AS1517499 and GW9662 evidently abrogated the suppressive effect of rTsDPP1 on CD64 expression relative to rTsDPP1 alone (*F =* 67.58, *P <* 0.0001) (Figure 15A). The rTsDPP1-suppressed cytotoxicity of ADCC killing NBL was also obviously regained by using AS1517499 and GW9662 compared to the rTsDPP1 alone group (*F =* 36.41, *P* < 0.0001) (Figure 15B). The NBL in the rTsDPP1 group had stronger motile ability, and fewer macrophages adhered to the NBL. However, in the AS1517499- and GW9662-treated macrophages, cytotoxicity was also enhanced, as demonstrated by the weakened NBL activity and increased number of macrophages attached to the NBL (Figure 16). The results suggested that the inhibitory effect of rTsDPP1 on macrophage FcγR I expression was involved in the activation of the STAT6/PPARγ pathway. Therefore, our results demonstrated that rTsDPP1 suppressed the mRNA expression of CD64 and inhibited macrophage-mediated ADCC, which was dependent on the activation of the STAT6/PPARγ pathway.

## Discussion

During *T. spiralis* infection, the host produces a strong immune response, which results in substantial damage to the host’s tissues and cells. To alleviate the damage in the host and to maintain long-term survival, which are necessary to create a balanced immune environment that benefits its parasitism, *T. spiralis* releases multiple immunoregulatory factors. Some studies have shown that *T. spiralis* ESA reduced the secretion of IL-1, IL-6, and IL-12 in LPS-stimulated macrophages and reduced the phosphorylation of ERK1/2 and p38 MAPK in macrophages. Additionally, ESA alone upregulated the expression of IL-10, TGF-β and Arg1 in macrophages [23, 55]. Recent studies revealed that AW ESA significantly upregulated the expression of CD206 and Arg-1 in macrophages and attenuated DSS-induced murine colitis by activating M2 macrophage polarization [24]. TsDPP1 was a highly expressed ESA of intestinal *T. spiralis* stages (IIL and AW), but its immune regulatory role in macrophages is largely unknown.

In the current study, to investigate the effect of rTsDPP1 on the polarization of RAW264.7 and mouse peritoneal macrophages, endotoxin-free rTsDPP1 was used to directly stimulate macrophages, and LPS served as the inducer of M1 and IL-4 as the inducer of M2. The AW ESA and TRX tag proteins of rTsDPP1 were used as positive and negative controls, respectively. The results of the CCK-8 assay showed that the appropriate concentration of rTsDPP1 to stimulate macrophages was 20 µg/mL; this dose had no obvious effects on cell viability. The results of IFT and confocal microscopy revealed that rTsDPP1 specifically bound to macrophages, and the binding site was localized on the cell membrane of macrophages, suggesting that there is an interaction between rTsDPP1 and host macrophages [39]. However, the TsDPP1-specific receptor on the surface of macrophages is still unclear, and it is necessary to further characterize the properties of TsDPP1-binding receptors on macrophages by coimmunoprecipitation, pull-down assays and mass spectrometry in future experiments.

To investigate the effect of rTsDPP1 on macrophage polarization, the expression of M1/M2 macrophages effector molecules was also measured in this study. The results revealed that rTsDPP1 induced high expression of Arg1 (M2-specific) in RAW264.7 macrophages but had no obvious effects on the expression level of iNOS (M1-specific), indicating that rTsDPP1 promoted M2 polarization in RAW264.7 macrophages. Moreover, the transcript levels of M2 genes (TGF-β, IL-10, CD206 and Arg1) and the surface molecule CD206 were found to be significantly increased in rTsDPP1-treated macrophages. The consistent immunomodulatory effects of rTsDPP1 on macrophage polarization were further confirmed in murine peritoneal macrophages. In contrast, the TRX tag protein had no evident effect on macrophage polarization. The results indicated that rTsDPP1 had the capacity to directly polarize macrophages to an M2 phenotype in vitro, and that peritoneal macrophages possessed the same ability to be polarized to the M2 phenotype and produce cytokines as RAW264.7 cells [56]. These findings are in accordance with the macrophage M2 polarization in *T. spiralis* infection and in after stimulation with AW ES products [24, 57]. Anti-inflammatory cytokines (TGF-β and IL-10) secreted by M2 macrophages were crucial to suppress excessive inflammatory reactions during *Trichinella* infection.

IL-4 and IL-13 are produced during helminth infections, which in turn activates the IL-4R/STAT signalling pathway [58]. After binding with IL-4Rα, a receptor on the surface of macrophages, IL-4 activates the intracellular tyrosine kinase JAK1 and further activates the downstream protein STAT6. Phosphorylated STAT6 binds to PPARγ and enters the cell nucleus to upregulate the expression of M2-type macrophage-related genes [59]. However, *T. spiralis* infection induces M2-type macrophage polarization in IL-4R-deficient mice, and this process is still dependent on STAT, suggesting that STAT is a key signalling molecule in M2 macrophage polarization [33]. In the present study, our results revealed that the expression levels of p-STAT6, STAT6 and PPARγ were significantly increased in rTsDPP1-treated peritoneal macrophages, suggesting that the mechanism by which rTsDPP1 activates M2-type macrophages is also dependent on the STAT6/PPARγ pathway. To further verify this hypothesis, a STAT6 inhibitor (AS1517499) and PPARγ antagonist (GW9662) were used to inhibit the effect of rTsDPP1-induced macrophage polarization in this study. The results showed that the two inhibitors abrogated the role of rTsDPP1 in inducing M2 polarization, as demonstrated by the fact that rTsDPP1 induced high expression of p-STAT6, STAT6 and PPARγ and that the high transcript levels of the TGF-β, IL-10, CD206 and Arg1 genes were evidently abrogated by the two inhibitors. The results indicated that the effect of rTsDPP1 on inducing macrophage M2 polarization was dependent on the activation of the STAT6/PPARγ pathway [50].

Macrophages, as a main effector cell, can directly kill and destroy *T. spiralis* NBL by producing and releasing NO and mediating the function of ADCC [21]. In this study, the release of NO in rTsDPP1-treated macrophages preinduced by LPS was also assayed. The results showed that rTsDPP1 reduced the NO synthesis and secretion by macrophages stimulated by LPS due to its activation of macrophage M2 polarization. FcR I (CD64), a receptor that binds to the antibody IgG Fc fragment on the surface of macrophages, is a key surface molecule that enables macrophages to mediate ADCC [60]. The flow cytometry results found that the mean fluorescence intensity of CD64 protein in macrophages stimulated by rTsDPP1 was distinctly reduced, and the qPCR results also showed that the transcript level of the CD64 gene was overtly decreased. Consequently, the larval killing capacity of rTsDPP1-treated macrophages via ADCC was also significantly reduced. Moreover, the rTsDPP1 inhibitory role on CD64 expression and the cytotoxicity of macrophages was clearly abrogated by using a STAT6 inhibitor and PPARγ antagonist, further suggesting that the rTsDPP1 inhibitory role was also involved in the activation of the STAT6/PPARγ pathway.

In conclusion, our results showed that rTsDPP1 had the ability to upregulate the expression of Arg1 and CD206 and increase the transcript levels of the TGF-β, IL-10, CD206 and Arg1 genes, which drove macrophages to the M2 phenotype by activating the STAT6/PPARγ pathway. The synthesis and release of NO were obviously reduced in rTsDPP1-activated M2 macrophages. Moreover, the cytotoxicity and larval killing capacity of rTsDPP1-induced M2 polarized macrophages via ADCC was also significantly reduced due to the downregulated expression of FcR I (CD64) and NO. The function of rTsDPP1 in promoting macrophage M2 polarization might contribute to the production of the immunosuppressive factors TGF-β and IL-10, which could regulate the host’s inflammatory responses to *T. spiralis* infection. This anti-inflammatory process might be beneficial to the parasitism and immune evasion of the nematode. The results of this study provide novel insights to further understand the mechanisms of immunomodulation and immune evasion by *Trichinella* during infection.

## Abbreviations

ML: muscle larvae
IIL: intestine infective larvae
AW: adult worms
NBL: newborn larvae
ESA: excretory-secretory antigens
M1: classical activated macrophage
M2: alternative activated macrophage
LPS: lipopolysaccharide
rTsDPP1: recombinant *T. spiralis* dipeptidyl peptidase I
ADCC: antibody-dependent cell-mediated cytotoxicity
FcR: Fc receptor
NO: nitric oxide
dpi: days post-infection
PEC: peritoneal exudate cells
TRX: thioredoxin
IFT: immunofluorescence test
DAPI: 4′,6-Diamidino-2-phenylindole
PMSF: phenylmethylsulfonyl fluoride
PVDF: polyvinylidene difluoride
TBST: Tris-buffered saline Tween
HRP: horseradish peroxidase
qPCR: real-time quantitative PCR
FSC-A: forwards scatter-area
FSC-H: forwards scatter-height
